# Molecular basis of ageing in chronic metabolic diseases

**DOI:** 10.1007/s40618-020-01255-z

**Published:** 2020-05-01

**Authors:** R. Spinelli, L. Parrillo, M. Longo, P. Florese, A. Desiderio, F. Zatterale, C. Miele, G. Alexander Raciti, F. Beguinot

**Affiliations:** 1 Department of Translation Medicine, Federico II University of Naples, 80131 Naples, Italy; 2 URT Genomic of Diabetes, Institute of Experimental Endocrinology and Oncology, National Research Council, 80131 Naples, Italy

**Keywords:** Ageing, Cellular senescence, Adipose tissue, Obesity, Type 2 diabetes, DNA methylation

## Abstract

**Aim:**

Over the last decades, the shift in age distribution towards older ages and the progressive ageing which has occurred in most populations have been paralleled by a global epidemic of obesity and its related metabolic disorders, primarily, type 2 diabetes (T2D). Dysfunction of the adipose tissue (AT) is widely recognized as a significant hallmark of the ageing process that, in turn, results in systemic metabolic alterations. These include insulin resistance, accumulation of ectopic lipids and chronic inflammation, which are responsible for an elevated risk of obesity and T2D onset associated to ageing. On the other hand, obesity and T2D, the paradigms of AT dysfunction, share many physiological characteristics with the ageing process, such as an increased burden of senescent cells and epigenetic alterations. Thus, these chronic metabolic disorders may represent a state of accelerated ageing.

**Materials and methods:**

A more precise explanation of the fundamental ageing mechanisms that occur in AT and a deeper understanding of their role in the interplay between accelerated ageing and AT dysfunction can be a fundamental leap towards novel therapies that address the causes, not just the symptoms, of obesity and T2D, utilizing strategies that target either senescent cells or DNA methylation.

**Results:**

In this review, we summarize the current knowledge of the pathways that lead to AT dysfunction in the chronological ageing process as well as the pathophysiology of obesity and T2D, emphasizing the critical role of cellular senescence and DNA methylation.

**Conclusion:**

Finally, we highlight the need for further research focused on targeting these mechanisms.

## Introduction

Human life expectancy has increased at a very rapid rate. Over the past 200 years, the mean age to death for countries with the most extended lifespans has steadily risen by 2.5 years per decade [1]. Nevertheless, the quality of life for elderly individuals did not proportionately increase. Indeed, although ageing may occur even in the absence of diseases (e.g., centenarians), ageing is also the major risk factor for most disorders with a significant public health impact [1–3]. These are known as age-related diseases (ARDs) and include chronic metabolic disorders such as obesity, type 2 diabetes (T2D), and cardiovascular disease (CVD), as well as cancer, neurodegenerative diseases and kidney diseases [1]. Interestingly, many ARDs, including obesity and T2D, show feature mimicking accelerated ageing at a younger age [1, 4]. Obesity per se conveys a higher risk of T2D, and both of them are associated with a premature onset of other ARDs, including CVDs and cancer [1, 4–9]. Ageing and chronic diseases may, therefore, share common pathophysiological mechanisms. In accordance with this view, we describe a spectrum of phenotypes driven by a common set of molecular and cellular pathways where the extremes are defined by centenarians, who have survived most ARDs and are distinguished by decelerated ageing, and by individuals who are prematurely affected by one or more ARDs and show signs of accelerated ageing [10]. As such, ageing and ARDs may be viewed as alternative trajectories of the same process that takes place at different rates, based on interactions between genetic, epigenetic and environmental factors, and lifestyle throughout the lifespan [10].

**Fig. 1:**
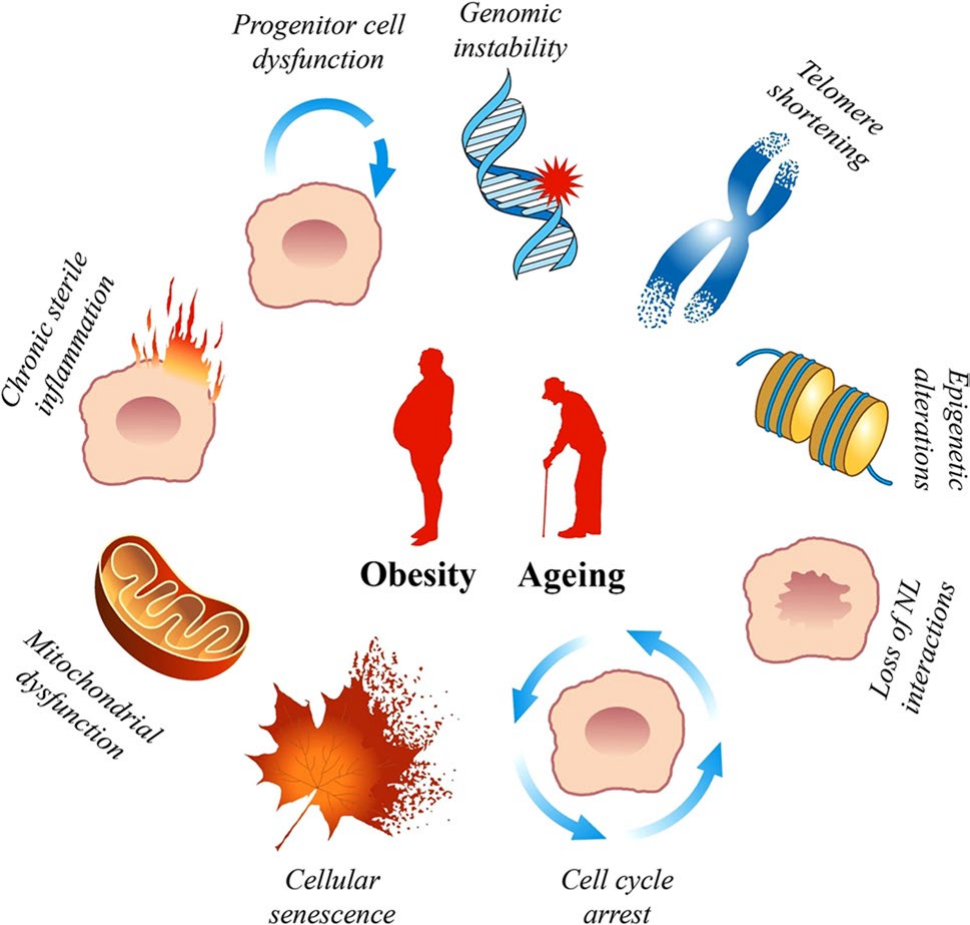
Molecular and cellular hallmarks of ageing. These hallmarks recapitulate the most remarkable features of ageing and depict the mechanisms underlying the pathogenesis of age-related diseases, including obesity and type 2 diabetes. NL, nuclear lamina

The mechanisms shared by ageing and ARDs can be grouped into the following processes: (i) decline in progenitor cell function; (ii) cellular senescence; (iii) chronic sterile inflammation; and, (iv) dysfunction of the macromolecular and cell organelles (e.g., genomic instability, shortening of telomeres, epigenetic changes, loss of the nuclear lamina interactions, and mitochondrial dysfunction) (Fig. 1) [2].

Most chronic diseases are defined, at least in part, by one or more of these pathways that affect the tissues specifically involved in the development of the pathological condition, e.g. adipose tissue (AT) in patients with obesity and/or T2D [1–5, 11, 12]. Any single ageing mechanism activation tends to influence others. Therapies targeting at the above-mentioned mechanisms may prevent, delay or mitigate multiple ARDs as a group and increase the healthspan [1].

Here, we will review the ageing pathways that contribute to obesity and T2D progression, emphasizing the causal link between age-related AT dysfunction and the systemic declines that lead to diabetes onset.

In this scenario, we will discuss how chronological as well as accelerated ageing occurs in AT, focusing primarily on cellular senescence. Finally, we will address the DNA methylation events that have recently been shown to impact on the ageing process.

## AT aging: an overview

AT is a highly dynamic endocrine and immune organ that plays a crucial role in controlling systemic metabolic homeostasis and inflammation. It undergoes complex changes in cellularity, insulin response, secretion profile, and inflammation during ageing with a dramatic impact on fat mass and distribution. Without intervention, these alterations lead to an elevated age-related threat of chronic metabolic diseases, with profound effects on health and longevity [4, 5, 13, 14]. Throughout early life, by controlling adipocyte progenitor cell (APC) differentiation and fat cell turnover, AT can efficiently respond to a wide range of changes in energy supply and regional microenvironment. The capacity to cause this compensatory response differs by local fat distribution, e.g. subcutaneous adipose tissue (SAT) and visceral adipose tissue (VAT) [5]. Molecular, cellular, physiological, and anatomical differences between SAT and VAT highlight the specificity of each fat depot and its characteristic function [15].

Nevertheless, due to the large replicative and adipogenic capacity of APCs and the decreased lipolytic activity and lower insulin sensitivity of adipocytes in SAT compared to those in VAT, subcutaneous fat can expand by increasing adipocyte cell size (hypertrophy) and number (hyperplasia), while visceral fat typically expands by increasing adipocyte cell size [16]. As such, SAT is well suited for long-term storage of lipids in response to a positive energy balance. Thus, surplus free fatty acids (FFAs) are stored in adipocytes as triglycerides, guarding against lipotoxicity in other tissues. Once this storage capacity is exceeded and the ability to develop new adipocytes is compromised, SAT becomes hypertrophic, inflamed, and dysfunctional, and surplus lipids start to accumulate in other AT depots (i.e., VAT or peri/epicardial fat) and in ectopic locations (i.e., liver and skeletal muscle). These events lead to local and systemic inflammation and insulin resistance (IR), which in turn contribute to the onset of T2D [17]. SAT adipocyte hypertrophy commonly occurs in elderly individuals, obese patients, and persons with a close family history of T2D, i.e. first-degree relatives (FDRs), and has been shown to represent an independent predictor for IR and a potential risk factor for T2D [18–20].

Many studies have described the dynamics of age-related changes in fat mass and regional distribution [21–23]. Redistribution of fat from subcutaneous to intra-abdominal visceral depots occurs primarily in men and women throughout middle age and is independent of changes in total adiposity, body weight, or waist circumference [4, 13, 23]. In elderly individuals, fat is stored outside of these AT depots and accumulates in muscle, liver, and other ectopic sites [4, 14, 19–23]. As a consequence, adipocyte hypertrophy, inflammation, and fibrosis arise in SAT during the early stages of ageing before glucose tolerance is impaired and local IR progressively develops. Indeed, systemic IR will not occur before advanced ageing [24]. Altogether, these metabolic disturbances contribute to the development of T2D and other ADRs, such as CVD and NAFLD [5].

In support of the pathophysiological significance of these age-related AT dysfunction, clinical studies in humans, including nonobese (i.e., lean or overweight) individuals, have clearly demonstrated that treatments affecting fat mass, such as calorie restriction (CR), exercise, and bariatric surgery, have beneficial effects on energy metabolism and metabolic risk factors for T2D, CVD, and cancer. The surgical removal of AT in humans, however, did not show the same metabolic effects as those caused by CR and exercise [25–28]. This may be linked to the ability of CR and exercise interventions to simultaneously reduce fat mass and promote AT remodelling by improving adipocyte turnover and formation [26–28]. In line with these observations, data from human and animal models suggest that intervention strategies proven to increase mean and maximal lifespan, such as single-gene manipulation (e.g., growth hormone/insulin-like growth factor 1 (GH/IGF-1) axis) and pharmacological and nutritional interventions (e.g., metformin, rapamycin, and CR) are useful in ameliorating age-related AT dysfunction [4, 25].

These data indicate that the cascade of molecular and cellular events underlying age-related AT damage starts in SAT and is caused by the reduced function of resident APCs, increased inflammation, and accumulation of senescent cells [4, 13, 14]. Telomere length (TL) erosion is known to represent a significant marker of ageing, reduced replicative ability, and senescence, both at the cellular and tissue levels [29]. Interestingly, age-related TL shortening mainly occurs in SAT compared to VAT and is due to shorter telomeres in the stromal vascular fraction (SVF) cells which include APCs. This evidence supports the concept that ageing of APCs is linked to compromised SAT hyperplastic/healthy expansion [30, 31].

Several studies indicate that age reduces the replicative and adipogenic capacity of SAT APCs [12, 32–35]. Accordingly, APCs isolated from SAT of healthy elderly subjects (age > 60) display decreased proliferation and differentiation capacities relative to APCs isolated from young individuals (age 18–30).

In these elderly subjects, the declining function of APCs is associated with high plasma levels of the inflammatory marker soluble tumour necrosis factor receptor 2 and increased AT secretion of the pro-inflammatory cytokine tumour necrosis factor-α (TNF-α) [34]. These findings support the notion that, during ageing, the progressive impairment in adipogenesis is related to a pro-inflammatory condition of SAT, which, in turn, contributes to limiting insulin sensitivity in the tissue.

Another process by which elderly people may be predisposed to IR is the “dysdifferentiation” of mesenchymal progenitor cells into partly differentiated adipocyte-like cells, i.e. mesenchymal adipocyte-like default (MAD) cells. MAD cells arise from APCs, which fail to complete differentiation into functional adipocytes. These were characterized by decreased sensitivity to insulin, irregular handling of fatty acids, and enhanced production of TNF-α [35, 36].

The causes of APC ageing are likely multifactorial and may include inherent genetic features of APCs and epigenetic factors, while the AT microenvironment can also play a role [13]. Current data underline a causal role for AT inflammation in this scenario [13, 14, 35]. High levels of pro-inflammatory cytokines and chemokines are found in both the fat tissue and blood of older adults. This condition, known as inflammageing, is a high-risk factor for many ARDs, multi-morbidity, and frailty [37]. AT is a progressively an increasing source of TNF-α, Interleukin-8 (IL-8), IL-6, IL-1β, and monocyte chemotactic protein-1 (MCP-1) in serum from ageing individuals [13, 14, 34, 37]. The circulating levels of these pro-inflammatory markers are also associated with the occurrence of T2D, their secretion is enhanced in SAT adipocytes of insulin-resistant individuals and positively correlates with the size of adipocytes in these same subjects [18, 38].

The finding that the age-related IL-6 increase is up to tenfold higher in SAT than in VAT indicates that inflammation associated with age is more severe in subcutaneous than in visceral fat [13]. The prevalent cell types responsible for age-related inflammatory changes in SAT are APCs and AT macrophages (ATMs) [35, 39–41]. During ageing, APCs release further pro-inflammatory chemoattractant factors (e.g., MCP-1), which promote the activation of ATMs. Upon activation, ATMs secrete pro-inflammatory cytokines which further enhance the inflammatory phenotype of both APCs and adipocytes [18]. The cross-talk between APCs, adipocytes, and ATMs creates self-perpetuating processes that maintain a pro-inflammatory milieu in SAT and drive chronic systemic inflammation, leading to metabolic dysfunction [35]. The underlying mechanisms of age-related inflammation are still far from being fully understood. Nevertheless, growing evidence from animal and human studies suggests a causal role of cellular senescence. Most senescent cells feature a senescence-associated secretory phenotype (SASP) which is characterized by a substantial increase in the secretion of pro-inflammatory factors. The SASP is a dynamic and complex phenotype composed of a wide range of cytokines, chemokines, proteases, and growth factors, which vary with cell type and cell microenvironment. Thus, senescent cells are a major contributor to the age-related pro-inflammatory AT secretion profile [42].

SAT is a crucial site for the accumulation of senescent cells during ageing. Indeed, age-related TL shortening occurs primarily in SVF cells isolated from SAT [42], also supporting the concept that APCs are among the more senescent-susceptible human progenitor cells [13]. Senescent APCs operate both in a cell-autonomous manner to enhance evolution toward senescence and to restrain adipogenic and lipogenic functions, and in a non-cell-autonomous manner to alter the tissue microenvironment by paracrine mechanisms. When secreted, SASP factors suppress adipocyte differentiation in neighbouring non-senescent APCs, cause inflammation of adjacent healthy cells and propagate senescence, inducing local and systemic detrimental effects even with low numbers of senescent cells [2, 43–47]. Accordingly, current evidence revealed that transplanting a small number of senescent murine or human APCs (i.e., 0.5–1 million) into middle-aged mice is sufficient to cause disability within 2 months, accelerate the onset of ARDs, and reduce survival. Only 2–10% of cells in AT are senescent in aged mice [48]. Similarly, the number of senescent cells is usually low in healthy middle-aged subjects (usually < 5%) and increases in several tissues, including AT, after 60 years of age (usually < 20%) [45]. Frailty, chronic diseases, and accelerated mortality appear to occur above a threshold number of senescent cells [43–48]. Similar findings were drawn from preclinical studies in mice (e.g., aged mice, progeroid mice, and diet-induced obese mice), and from human clinical trials (e.g., diabetic kidney disease individuals). Indeed, removing just 30% of senescent cells using either genetic or pharmacological strategies (i.e., senolytics and SASP inhibitors) was effective in preventing, alleviating, or reversing age-related dysfunctions, such as AT defects, inflammation, and IR [48–57].

Such findings underline the crucial role of senescent APCs in driving ageing phenotypes and strongly support the selective targeting of these cells as a novel way of alleviating chronic disorders of metabolism and increasing the duration of human health.

## Pathophysiological processes in age-related AT dysfunction

### APC function decline

APCs represent a large population of progenitor cells in human tissues, accounting for 15–50% of AT cells [13]. They undergo a wide range of functional changes during ageing [13], including a reduction in replicative capacity [34, 58], an impairment of adipogenesis [31, 34, 39, 58, 59], a decrease in the handling of fatty acids [60], and an increase in secretion of pro-inflammatory cytokines and chemokines, metalloproteases, and stress response elements [44, 45]. Notably, these age-related changes are maintained and are still detectable ex vivo in cultured AT APCs from animal and human donors of different ages. In addition, they are often reflected in whole fat tissue and/or isolated adipocytes [31, 32, 44, 45, 58–60].

Extensive studies have shown that APCs obtained from older donors have reduced lipid accumulation capacity after induction of adipocyte differentiation compared to APCs from their younger counterparts [34, 39, 59, 61]. Age-related restricted adipogenesis is due, at least in part, to the altered expression of the adipogenic transcription factors CCAAT/enhancer-binding protein α (C/EBPα) and peroxisome proliferator-activated receptor γ (PPARγ) [13, 59]. C/EBPα and PPARγ have essential roles in initiating differentiation programmes and cooperate in controlling the expression of genes needed to acquire and sustain adipocyte phenotypes [62, 63].* C/ebpα* mutant mice feature a progeroid phenotype showing lifespan reduction and alterations in body weight, fat depots, and glucose homeostasis [59]. Similarly, lowered *Pparγ *expression in mice causes lifespan decline and lipodystrophy [64]. In both APCs and whole AT, the expression of C/EBPα and PPARγ decreases with age [34, 35, 39, 58, 59, 61]. Consistently, C/EBPα overexpression restored the adipogenic potential of APCs isolated from older adults [59]. Anti-adipogenic regulators, including C/EBPβ liver inhibitory protein (C/EBPβ-LIP), C⁄EBP homologous protein (CHOP), and CUG triplet repeat-binding protein (CUGBP), are also altered during ageing [59, 63]. The expression levels of C/EBPβ-LIP, CHOP, and CUGBP are increased in APCs, adipocytes, and intact AT during ageing [58, 59, 61].

Since several genes downstream of C/EBPα and PPARγ modulate insulin sensitivity, fatty acid handling, and mitochondrial function, age-related adipogenic impairment contributes to altered fatty acid β-oxidation, lipotoxicity, and IR [59–61].

All of these molecular and cellular abnormalities appear at different rates in SAT and VAT, and SAT appears to be mainly affected [13]. The differences in gene expression profile, epigenetic pattern, and exogenous microenvironment between these fat depots are responsible for their specific susceptibility to age-related changes [65, 66].

### Chronic sterile inflammation

Sterile inflammation develops without external causes (e.g., infection), and chronic sterile inflammation is a feature of ageing and is more pronounced in AT [37]. During ageing, the AT secretory profile shifts to a more pro-inflammatory signature in response to physical, chemical, or metabolic stimuli (e.g., genomic, hypoxic, nutrient, oxidative, and endoplasmic reticulum (ER) stress) [4, 13, 14, 35, 37]. APCs predominantly secrete more pro-inflammatory cytokines and chemokines that trigger a cascade of events driving the surrounding cells to an inflammatory state. This, in turn, results in derangements in adipocyte function, activation of ATMs, and recruitment of T-lymphocytes and monocytes from blood [35, 36].

Human studies indicate that expression and secretion of TNF-α, IL-6, and MCP-1 are higher in APCs from elderly subjects than in those from younger individuals and that ATM content in healthy subjects is positively correlated with age [13, 14, 34, 37, 41]. Analysis of age-related inflammatory and immune changes in murine AT has shown that ER stress plays a central role in controlling APC and ATM inflammatory responses. Indeed, decreasing ER pressure through chemical chaperones in aged SVF cells and ATMs lowers the concentrations of TNF-α, IL-6, and MCP-1, both in vitro and in vivo [67–69].

During ageing, the AT T-cell population is also modified. The number of regulatory T cells (Tregs) is increased by threefold in aged AT. The higher Treg activation correlates with age-related epigenetic drift and hypomethylation of T-cell DNA and leads to age-related immune deficiency predisposition [70–72]. Interestingly, selective depletion of fat-specific Tregs attenuates several symptoms of age-related metabolic dysfunction in rodents, including IR. Notably, Tregs accumulate in AT as a result of age but not obesity. Thus, age- and obesity-associated IR can involve distinct AT immune populations [73].

As mentioned above, APC inflammation markers in SAT were higher than in VAT [14]. Since SAT is 10 to 20 times more abundant than intra-abdominal VAT [35], the inflammatory and immune changes correlated with age in this fat depot may have significant systemic effect on metabolism.

### Cellular senescence

In adult tissues, senescence is transiently induced as a response to injury to maintain homeostasis by removing damaged cells. Senescent cells, however, may persist and accumulate in tissues during ageing due to immune system deficiencies (i.e., immunosenescence) [74]. Persistent senescent cells render senescent cell-rich tissues less functional and more susceptible to stressors, interfering with the outcome of several physiological and pathological processes. While all types of cells undergo senescence, some are more susceptible than others [75, 76]. Data from both human progeria syndromes and progeroid mouse models indicate that APCs are highly prone to senescence, supporting the concept that APC senescence determines age-related AT dysfunction [76–78].

Cellular senescence has been identified as a response to different stressors (depending on the in vivo context) which converge on activation of common effectors [76]. Many of these stimuli are signalled by pathways that overlap in activating the tumour suppressor protein 53 (p53) and upregulate the cyclin-dependent kinase (CDK) inhibitors p21 and p16. The inhibition of the CDK-cyclin complex results in permanent cell cycle arrest, a characteristic feature of senescent cells [79]. When growth arrest occurs as a consequence of telomere erosion following multiple cell divisions, senescence is termed replicative senescence. Instead, when the arrest of the cell cycle is independent of TL shortening, senescence is termed stress-induced premature senescence [80]. DNA damage, oncogene activation (e.g., Ras), oxidizing agents and metabolites (e.g., H_2_O_2_), mitochondrial dysfunction, epigenetic changes (e.g., methylation of DNA) and paracrine SASP factors may induce growth arrest [81].

SASP acquisition is downstream of senescence induction and may confer pleiotropic functions to senescent cells. The SASP is highly heterogeneous and controlled at multiple levels [82]. At the transcriptional level, it is mainly controlled by nuclear factor-kB (NF-kB), which primarily regulates the production of pro-inflammatory cytokines and chemokines [82]. Upstream, Janus kinase (JAK), p38, and other MAP kinases jointly control SASP expression [74, 81, 82]. Moreover, epigenetic changes fine-tune SASP genes by keeping their chromatin loci and control regions in an open and active state [82]. The main pathway involved in SASP protein secretion is the mechanistic target of rapamycin (mTOR) signalling network [81, 82].

SASP actions are multiple, and their effects are tissue and context specific. Indeed, they depend on (i) the nature of the SASP secretome; (ii) the inherent genetic and epigenetic proprieties of the cells exposed to it; and, (iii) the surrounding microenvironment [75]. IL-8, IL-6, and transforming growth factor β-1 (TGFβ-1) are among the specific factors by which the SASP reinforces and spreads senescence with both autocrine and paracrine mechanisms [74]. Since subcutaneous APCs are more vulnerable to senescence, SASP acquisition is also relevant for the age-related limited hyperplastic expansion and storage capacity of SAT [2, 15]. Indeed, among the SASP components released by senescent APCs IL-6, TNF-α, interferon-γ (IFN-γ), and activin A can directly impair both adipocyte differentiation and insulin sensitivity [44]. Senescent APCs can recruit immune cells to AT by secreting the SASP factors IL-6, IL-8, MCP-1, and plasminogen activator-1 (PAI-1) [45].

The use of both senolytic drugs and genetic models for senescence ablation confirmed the causal role of senescent APCs in age-related AT metabolic dysfunction and inflammation. Targeting senescent cells by SASP inhibition in old mice is effective in enhancing adipogenic and metabolic functions and decreasing AT and systemic inflammation [44–46]. Additional evidence for this concept arises from studies in mice lacking for telomerase (*Tert*), which feature shorter telomeres with successive generations. Fourth-generation (G4) *Tert-*knockout mice express high levels of senescence markers and are characterized by macrophage infiltration in AT, glucose intolerance, and IR. Interestingly, surgical removal of AT in G4 *Tert*-knockout mice improves glucose metabolism, whereas transplantation of AT from G4 *Tert*-deficient mice into age-matched wild-type mice causes IR. This effect is attenuated by transplanting wild-type mice with AT deficient for both *Tert *and *p53*, thereby highlighting a crucial role of p53 in development of IR [76]. These data are also consistent with recent evidence in humans that both donor age and subcutaneous adipocyte cell size are positively correlated with p53 expression in SAT, regardless of obesity. Thus, p53 can contribute to the age-related IR in humans [83].

Senescent cells exhibit typical structural and molecular changes as a result of the activation of the signalling pathways mentioned above, including acquisition of the enlarged and flattened cell body, activity of senescence-associated β-galactosidase (SA-β-gal), destabilization of nuclear integrity, and reorganization of chromatin [81]. Notably, the senescence-associated impairment of nuclear integrity is due to the downregulation of the *Lamin B1* (*LMNB1*) gene. The downregulation of *LMNB1* in senescent cells is a key trigger of global reconfiguration of chromatin which assumes a more open organization [84]. Although senescent cells feature these characteristics, they are not necessarily displayed simultaneously and with similar intensity, making the phenotype of senescence extraordinarily dynamic and complex. This characteristic, along with the possibility of finding specific senescence-associated traits outside of senescence, illustrates the potential unreliability of using a single marker to recognize senescent cells, especially in vivo. At the moment, search for a precise and sensitive senescence signature is ongoing.

The current best methods to identify senescent cells are based upon the use of combinations of SA-β-gal activity, upregulation of p16, and/or p21, expression of the strongly induced SASP genes, and downregulation of *LMNB1* [85]. Such marker combinations allow an accurate detection and efficient quantification of senescent cells both in vivo and ex vivo. This approach can be used to test the effectiveness of intervention strategies aimed at targeting senescent cells. Indeed, both heterochronic parabiosis studies, in which young (4 months) and old (20 months) mice are surgically linked to share circulation, and ex vivo treatment of SVF cells from young and old mice with young or old serum, have shown that a young milieu is effective in protecting old AT from senescence by reducing the levels of p16, p21, and pro-inflammatory SASP factors [86].

## Linking AT senescence to chronic metabolic diseases

### Obesity

Obesity is a major risk factor for IR and T2D. Obese people exhibit IR at a younger age compared to lean individuals, predisposing them to develop T2D [4, 87]. This early-onset IR is attributed to AT dysfunction and low-grade chronic inflammation, similar as in ageing [87]. The premature accumulation of senescent cells in AT represents a determining factor in linking obesity, ageing, AT dysfunction, and inflammation [44, 53, 79]. Indeed, selective removal of these cells from AT in obese mice alleviates the obesity-related derangement in fat tissue function and glucose homeostasis [53]. Accordingly, approaches that are successful in counteracting obesity and ageing, such as exercise and nutritional interventions, exert their health effects by targeting cellular senescence in AT [25, 26, 52, 87]. As epigenetic factors respond adaptively to lifestyle they may be implicated independently of age in the acquisition of a senescent phenotype [51, 88–93].

Animal models have been widely used to depict the mechanisms by which obesity promotes AT ageing and IR [87]. In the obesity setting, due to caloric overload, fat tissue is subjected to mechanical, hypoxic, oxidative, and ER stress. Once activated, the stress responses initiate a cascade of events in AT leading to senescence induction, functional decline, macrophage infiltration, and inflammation, resulting in IR [79, 87]. The harmful effects of excess nutrients and the protective influence of exercise in obesity-related AT ageing have been demonstrated by the use of middle-aged mice undergoing physical exercise and/or fast-food diet (FFD) feeding. Administration of a FFD simultaneously causes adverse effects on body weight and insulin sensitivity and increases the expression of senescence markers (i.e., SA-β-gal, p53, p21, and p16) and SASP factors (i.e., IL-6, MCP-1, and PAI-1) in fat tissue. By preventing senescent cell accumulation and SASP development, physical exercise neutralizes the FFD-induced detrimental effects on metabolic parameters [52].

The expression of p53 in AT plays a vital role in the development of obesity-related IR [87, 94]. P53 acts both as a potent senescence inducer and an adipogenesis repressor [83]. Indeed, it needs to be downregulated before APCs can differentiate into insulin-responsive adipocytes [95]. Furthermore, the activation of p53 in adipocytes impairs insulin-stimulated glucose transport, enhances lipolysis, and promotes inflammation [96]. Minamino et al*.* elucidated the role of p53 in linking obesity, AT senescence, and metabolic dysfunction [94]. Excessive caloric intake induces AT senescence, inflammation, and IR in agouti mice, a widely used model adopted to study nutritionally induced epigenetic effects on the obesity phenotype [89]. Interestingly, the adipocyte-specific p53 deficiency in the obese agouti mice exposed to a standard chow diet, as well as in obese wild-type mice fed a high-sugar/high-fat diet, is sufficient to decrease the expression of senescence and inflammatory markers in AT and to improve insulin sensitivity [94]. Therefore, cellular senescence and inflammation, due to high p53 levels in AT, lead to metabolic complications associated with obesity [13]. There is evidence that similar processes in obese patients are also activated in AT. Obesity is associated to increased AT expression of senescence markers in young/middle-aged subjects, including p53 [13, 79, 83]. Additionally, Justice et al*.* recently provided evidence of the effectiveness of a 5-month resistance training programme with or without CR in lowering these senescence markers in thigh AT in overweight/obese women [97].

Another emerging mechanism linking obesity and ageing to the development of IR is downregulation of the Sirtuin (SIRT) pathway [98]. Sirtuins act primarily as nicotinamide adenine dinucleotide (NAD)-dependent deacetylases [98], and SIRT1 is the most well-studied Sirtuin with effects on metabolism [99]. Mice lacking the *SIRT1* gene develop obesity and IR and have ectopic lipid accumulation, as well as increased AT inflammation when fed a HFD [100]. Consistently, adipocyte-specific *SIRT1* knockout mice show increased adiposity and are prone to become insulin resistant. When combined with HFD or ageing, the adipocyte-specific *SIRT1* depletion worsens IR by rising the number of ATMs and their polarization towards the pro-inflammatory M1 subtype [101–103]. Similarly, the macrophage-specific deletion of *SIRT1* enhances HFD-induced macrophage infiltration and inflammation of AT with concomitant worsening of IR [102–104]. Interestingly, the levels of SIRT1 decrease in mice upon diet-induced obesity, as well as during ageing [99]. Obese individuals also show reduced SIRT1 expression in SAT and VAT [105–107], as well as in both adipocytes and SVF cells isolated from the aforementioned fat depots [106]. There is a negative correlation between the AT *SIRT1* mRNA levels and homeostasis model assessment of insulin resistance (HOMA-IR) [106]. In addition, *SIRT1* expression in SAT is inversely correlated with BMI and ATM content [106, 107], while its expression in VAT is negatively correlated with BMI and waist circumference [105, 106]. This is consistent with the observation that obese adolescents with a high ratio of visceral to subcutaneous fat exhibit impaired *SIRT1* expression and increased macrophage content in SAT compared to BMI-matched subjects with a low-ratio visceral to subcutaneous fat [108]. Taking into account the functions of SIRT1 as both an inducer of adipogenesis in human visceral APC and a repressor of inflammatory pathways, its downregulation fosters VAT expansion and heightens the inflammatory state of AT in obesity, contributing to IR development [101, 102, 105, 109]. Notably, the deregulation of SIRT1 under caloric overload may also impact on p53 activity, exacerbating AT senescence [110–114]. Indeed, many studies, both in vitro and in vivo, have shown that SIRT1 deacetylates p53 and inhibits its transactivation, antagonizing the premature senescence induction [111]. However, p53 transcriptionally suppresses SIRT1 and induces the expression of miR-34 which binds to the 3′-UTR of the SIRT1 mRNA, blocking its translation [112, 115]. Thus, in the context of obesity, perturbations of interplay between SIRT1 and p53 appear to represent a key factor for the establishment of a vicious circle between AT senescence and inflammation that worsens IR.

Senescent APCs from SAT of obese individuals show a loss of replicative and differentiation ability [13, 79, 83, 116, 117]. Impaired SAT adipogenesis is a significant feature of hypertrophic obesity and is associated with IR and a very high risk of developing T2D [18]. The aetiological significance of this association was underlined by data from FDRs showing signs of both limited adipogenesis and adipocyte hypertrophy in SAT and IR even before becoming obese and elderly [18]. A large-scale genome-wide association study (GWAS) revealed that individuals with risk genes for T2D and IR phenotypes have limited capacity to expand the peripheral SAT compartments adequately [118]. Recently, Gustafson et al*.* provided a mechanistic explanation for the above correlation by showing that increased APC senescence is responsible for impaired SAT adipogenesis in hypertrophic obesity [83]. This study has revealed that senescence markers (i.e., SA-β-gal, p53, and p16) and SASP factors that antagonize differentiation (i.e., PAI-1 and TGFβ-1) are upregulated in SAT biopsies from patients with hypertrophic obesity and T2D and are positively correlated with subcutaneous adipocyte cell size [83].

Interestingly, the impaired adipogenic capacity of APCs isolated from the same specimens of SAT biopsy is linked to the inability to suppress p53 after induction of differentiation [83]. The evidence that a similar dysregulated pattern of increased p53 is also a feature of poorly differentiated APCs from SAT of young lean and healthy FDRs suggest that the premature senescence of APCs may contribute to the SAT dysfunction and metabolic abnormalities associated with an epi-/genetic predisposition for T2D [83]. As such, epi-/genetic factors associated with a family history of T2D may contribute to the FDR-associated APC phenotypes of both impaired adipogenesis [119] and premature senescence [120]. In this scenario, in-depth studies focusing on the epigenetic regulation of p53 and its effectors are of crucial importance.

### Type 2 diabetes

There is a dynamic relationship between T2D and senescence [121–128]. T2D in itself has been proposed to represent a state of accelerated ageing in which senescent cells are part of a pathogenic loop, both as a contributing cause and as a result of the metabolic disturbances observed in the prediabetic and diabetic states [121, 122]. Many GWASs also showed that single-nucleotide polymorphisms (SNPs) in genes encoding senescence markers, including p53, p16, and p21, are associated with an increased risk of developing T2D and its complications [123–125]. Notably, SNPs at p53 transcriptional target genes, such as *TCF7L2*, *TP53INP1*, *ZMAT3*, and *SIRT1*, are also associated with T2D and its related traits [99, 125]. In addition, cross-sectional studies have shown that leukocyte TL is negatively correlated with HOMA-IR in non-T2D young/middle-aged adults, regardless of obesity [126, 127]. In addition, a large sample of Caucasian T2D patients demonstrated a strong association between the leucocyte TL and the presence and number of diabetic complications [128].

Focusing on AT, TL shortening is inversely correlated with adipocyte cell size in SAT not only in obese subjects with or without T2D but also in lean diabetic patients, indicating a strong relationship of cellular senescence to the unhealthy metabolic environment related to adipocyte hypertrophy [129]. This link is also supported by data from adipose-p53-transgenic mice expressing high levels of both p53 and p21 in AT [94]. In these mice, the upregulation of these senescence markers is sufficient to induce an inflammatory state that drives IR [94]. Consistently, individuals with T2D typically display an elevated senescent cell burden in AT, as demonstrated by the high expression levels of SA-β-gal, p53, p21, and pro-inflammatory SASP components (*e.g.*, IL-1α, IL-1β, IL-6, and TNF-α) [94].

**Fig. 2:**
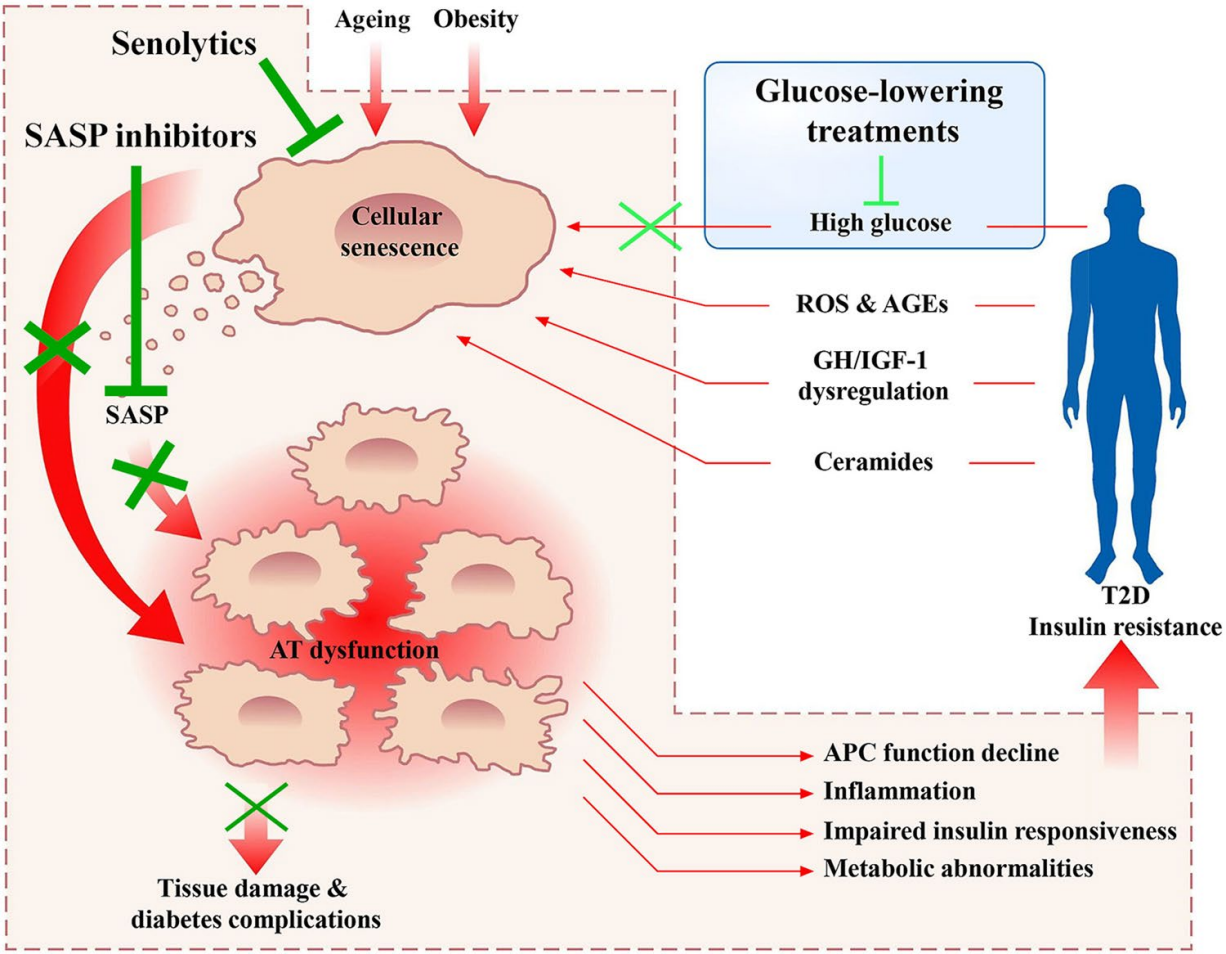
A vicious cycle explains the synergistic association between cellular senescence and type 2 diabetes. The increased senescent cell burden in adipose tissue during ageing and obesity contributes to inflammation, adipocyte progenitor cell dysfunction, impaired insulin responsiveness, and metabolic abnormalities. These effects promote to insulin resistance and type 2 diabetes. Metabolic, inflammatory, and immune perturbations in the diabetic state, in turn, fuel senescent cell accumulation, which contributes to tissue damage and diabetes-related complications. Senolytics and SASP inhibitors seem to be more effective (dark green blunt head arrow) in breaking this malignant positive feedback loop than current glucose-lowering treatments (light green blunt head arrow). *SASP* senescence-associated phenotype, *AT* adipose tissue, *T2D* type 2 diabetes, *APC* adipocyte progenitor cell, *ROS* reactive oxygen species, *AGEs* advanced glycation end products, *GH* growth hormone, *IGF-1* insulin-like growth factor-1

From a mechanistic perspective, cellular senescence and T2D form a vicious circle where both the obese and the prediabetic microenvironment become permissive for cellular senescence to develop prematurely. Senescence, in turn, exacerbates induction and impairs the clearance of senescent cells, resulting in tissue damage and metabolic derangement [121]. In this scenario, high glucose, GH/IGF-1 axis alterations, dyslipidaemia, low-grade chronic inflammation, and immune dysfunction are the most common triggers inducing senescence [121, 130] (Fig. 2).

High glucose levels may cause premature senescence in APCs as well as in other primary human cells (e.g., mesangial, endothelial, and renal cells), mainly by fostering mitochondrial dysfunction and accumulation of reactive oxygen species (ROS) [131–133]. The resulting increase in ROS production induces the activation of critical pathways implicated in diabetes-related complications, including the increased formation of advanced glycation end products (AGEs) [134, 135]. AGE signalling itself drives premature senescence [136]. p53-mediated premature senescence can result from chronic IGF-1 exposure, the increased levels of which are due to hyperinsulinaemia and the changes in IGF-binding proteins (IGFBPs) [137]. Among these, IGFBP3 has been recognized among the SASP components responsible for spreading senescence to bystander cells [138]. Ceramides act as a mediator of several types of stress responses that are primarily mediated by the p53 and p38 pathways. High ceramide levels can result in the senescence of adipose, endothelial, and immune cells due to alterations in the metabolism of fatty acids [139]. Altogether these events synergistically promote the accumulation of senescent cells and expression of the related SASP in AT. This event, in turn, drives the local and systemic inflammation and derangement of metabolic homeostasis, contributing to the onset of T2D.

Several studies on human cells from T2D subjects revealed that senescent cells are also spread within aetiological tissues involved in diabetes-related complications [121]. In particular, the kidneys of patients with type 2 diabetic nephropathy (DN) exhibit an accelerated senescent phenotype in selected cell populations, particularly tubular cells and podocytes, accounting for DN progression towards renal insufficiency and diabetic kidney disease (DKD) [140]. This hypothesis seems also confirmed by the latest interim report from a clinical trial in DKD patients evaluating a combination of senolytic medications, dasatinib plus quercetin (D + Q) [57]. Indeed, DKD patients treated with three daily doses of D + Q display a lower senescent cell burden in abdominal SAT (*i.e.*, decreases in SA-β-gal, p21, and p16) and lower plasma levels of the main SASP components (*e.g.*, IL-1α, IL-2, IL-6, and IL-9) within 11 days [57]. Since senolytics appear to be more effective in alleviating senescence-associated diabetes complications than the currently available glucose-lowering treatments, the opportunity to introduce these drugs into clinical practice may provide a new way to treat chronic diseases that are still untreatable [53, 57, 121] (Fig. 2). Importantly, previous investigations have shown that D + Q exposure eliminated senescent cells from aged mice, mice with IR and other chronic diseases, and AT explants from obese and/or diabetic patients [57, 141]. In additions, preclinical studies in HFD-induced or genetically obese (*db/db*) mice revealed that D + Q mitigated IR, proteinuria, and dysfunction of the renal podocytes by eliminating senescent cells, primarily senescent APCs, from AT [53].

## Epigenetics of ageing: a focus on DNA methylation

The processes of hormesis and adaptive homeostasis provide a possible explanation of why the same molecular mechanisms should drive healthy ageing and longevity on one side and unhealthy ageing and chronic metabolic diseases on the other. Lifelong exposures to low amounts of environmental stresses activate adaptive responses, making the organism better suited to the environment than before and providing benefits for ageing and health. However, the induced responses may determine detrimental effects when the dose, intensity, or duration of these stressors overcome the adaptive homeostasis capacity, which will accelerate the development of ageing and disease. Epigenetic changes are crucial processes behind these trajectories of alternative ageing [10, 142]. Indeed, age-related dysregulation of epigenetic control is a common aetiological factor for ARDs ranging from T2D to neurodegenerative diseases [143]. Moreover, many effective lifespan-extending interventions act through epigenetic pathways [142]. The opportunity to “reverse” ageing is an intriguing implication of epigenetic ageing regulation, which provides a mechanistic basis for evidence that ageing hallmarks can be reversed through parabiosis experiments [86].

By definition, epigenetic changes serve as heritable reversible mechanisms that modulate the functional use and stability of genetic information in response to environmental stimuli without modifying the underlying DNA sequence. These changes include DNA methylation, chromatin remodelling, histone modifications, and non-coding RNA transcription [89, 142–144]. Although each of these mechanisms is functionally relevant, geroscience research has best characterized the role of DNA methylation dynamics during ageing and their involvement in cellular senescence [142, 143]. DNA methylation mainly occurs at the 5′ position of the cytosine residues of cytidine-guanine dinucleotides (CpGs) and may be associated with either transcriptional repression or activation, depending on the site where it occurs. Generally, CpG methylation at promoters and enhancers causes gene silencing through chromatin condensation (i.e., heterochromatin). Conversely, a decrease in DNA methylation correlates with heterochromatin de-condensation and gene expression [89].

There is a global hypomethylation of CpG over the genome during ageing that is responsible for (i) loss of heterochromatin and gene de-repression in this region; (ii) nuclear architecture changes; and, (iii) increased genomic instability. Interestingly, the hypomethylation-induced loss of silencing at heterochromatic loci appears during ageing in all mammals, and its acceleration and rescue can, respectively, reduce and extend the lifespan [142]. In addition to global hypomethylation, the ageing process entails focal increases in DNA methylation at specific CpGs, resulting in heterochromatinization and gene silencing [142].

The age-associated genome-wide pattern in DNA methylation can be due to a progressive reduction in levels of DNA methyltransferases (DNMTs) and/or their critical substrates [e.g., S-adenosilmetionina (SAM)] [123, 126]. Indeed, age may alter the expression of DNMT1, DNMT3A, and DNMT3B in several human tissues (e.g., mononuclear peripheral blood cells and AT) and reduce the availability of SAM by affecting mitochondrial function [142, 145–147].

Paired methylome and transcriptome analyses in ageing cells and tissues from both mice and humans have revealed an inverse correlation between age-related differences in gene expression and DNA methylation [143, 147]. Quantitative measurements of these differences between young and old mammals show that they are in the range of 5–25% at susceptible genes [148]. Notably, these genes are enriched in pathways dysregulated during ageing such as senescence, inflammation, and the insulin-signalling pathway [143].

DNA methylation has been recognized as a critical mechanism promoting senescence and ageing at the molecular and cellular levels [149–153]. The DNA methylome of senescent cells shows extensive hypomethylation and formation of facultative heterochromatin domains compared to proliferating normal cells. In particular, bisulfite sequencing analysis of the methylome in both proliferating and senescent cells revealed a decline in CpG methylation in senescent cells ranging between 65.0% and 58.4%. Notably, these hypomethylation events are enriched in the lamin-associated domains which are dynamic regions that occupy up to 40% of the genome across different cell types and lead to the establishment and preservation of transcriptional microenvironments [153].

There is extensive evidence of the importance of DNA hypomethylation as a senescence inducer. First, this occurs in pre-senescent cells, but not in immortalized cells where the overall methylation level is relatively stable, indicating that DNA methylation dynamics are related to a limited proliferative lifetime [150, 153]. It was, therefore, postulated that DNA hypomethylation may function as a mitotic clock, similar to TL shortening [148]. Second, the use of DNMT inhibitors (e.g., 5-azacytidine) or specific small-interfering RNAs to target DNA methylation is sufficient to induce senescence in primary human cells [152]. Notably, senescence-related hypomethylation occurs predominantly in genes with reduced expression in proliferating cells but elevated expression in senescent cells [153]. These include genes that encode p53 targets *p21* and *p16*, as well as the two main SASP pro-inflammatory components *IL-6* and *IL-8* [82, 152]. Convergent findings support a model by which inflammation can directly and indirectly reduce DNA methylation [153–155]. Thus, by triggering chronic sterile inflammation, ageing and obesity can lead to a vicious cycle between senescence and DNA hypomethylation which helps explaining why T2D risk increases with age and BMI [148, 156].

Since DNA methylation plays a crucial role in determining senescence and linking several characteristics of ageing, it may represent a critical aspect of these processes. In line with this notion, many studies have shown that the pattern of DNA methylation in human tissues can be used as a chronological age estimator, a biomarker for healthy and unhealthy ageing, and a risk factor for ARDs [145]. AT has been extensively studied in this context supporting the notion that age-related changes to the methylome may underlie the AT dysfunction observed in the elderly population [147]. In particular, different groups have developed so-called DNA methylation clocks (DNAm-age clocks) based on age-associated DNA methylation changes that are relatively common across individuals and in some cases, across tissues [157–162]. Each clock utilizes DNA methylation information at specific CpGs (ranging between 3 and 353 CpGs) to calculate the time elapsed after birth (i.e., chronological age). Pathway analyses of the genes co-localizing with these CpGs reveal richness in biological processes correlated with tissue development, cell growth and proliferation, cell death and survival, and cancer [145, 163]. Notably, each DNAm-age clock has a high degree of accuracy, determined by the correlation coefficient between the actual chronological age and the expected chronological age (i.e., DNAm-age or epigenetic age) [145]. The two most analysed human clocks have correlation coefficients more than 0.9 of and a prediction performance error of less than 5 years [145, 157, 158].

Interestingly, several DNAm-age clocks investigated in vivo successfully predict in vitro chronological age. Their analysis shows that both systems display global hypomethylation throughout the human lifespan suggesting preservation of an epigenetic ageing signature between human tissues and primary human cells [164]. Notably, specific DNAm-age clocks also distinguish senescence from replicative states of cellular lifespan and are sensitive to environmental stimuli [145, 164, 165]. Accelerated rates of epigenetic ageing, both in vivo and in vitro, are correlated with metabolic stressors related to obesity and a shorter lifespan. Dietary factors are among the DNAm-age-related exposures.

Furthermore, insulin, glucose, triglycerides, and total cholesterol serum concentrations are positively correlated with DNAm-age acceleration. Several of these associations were found using evidence from 4173 women in the Women's Health Initiative study and 402 subjects in the European InCHIANTI study [163]. Consistently, ex vivo experiments have shown that human fibroblasts cultured under chronic hyperglycaemic conditions show an increase in the baseline DNAm-age of approximately 3 years [164]. Overall, these findings indicate that the longitudinal quantification of the epigenetic ageing rate in primary human cells can be a valid system for targeting or evaluating lifespan-wide age-modifying interventions.

**Fig. 3:**
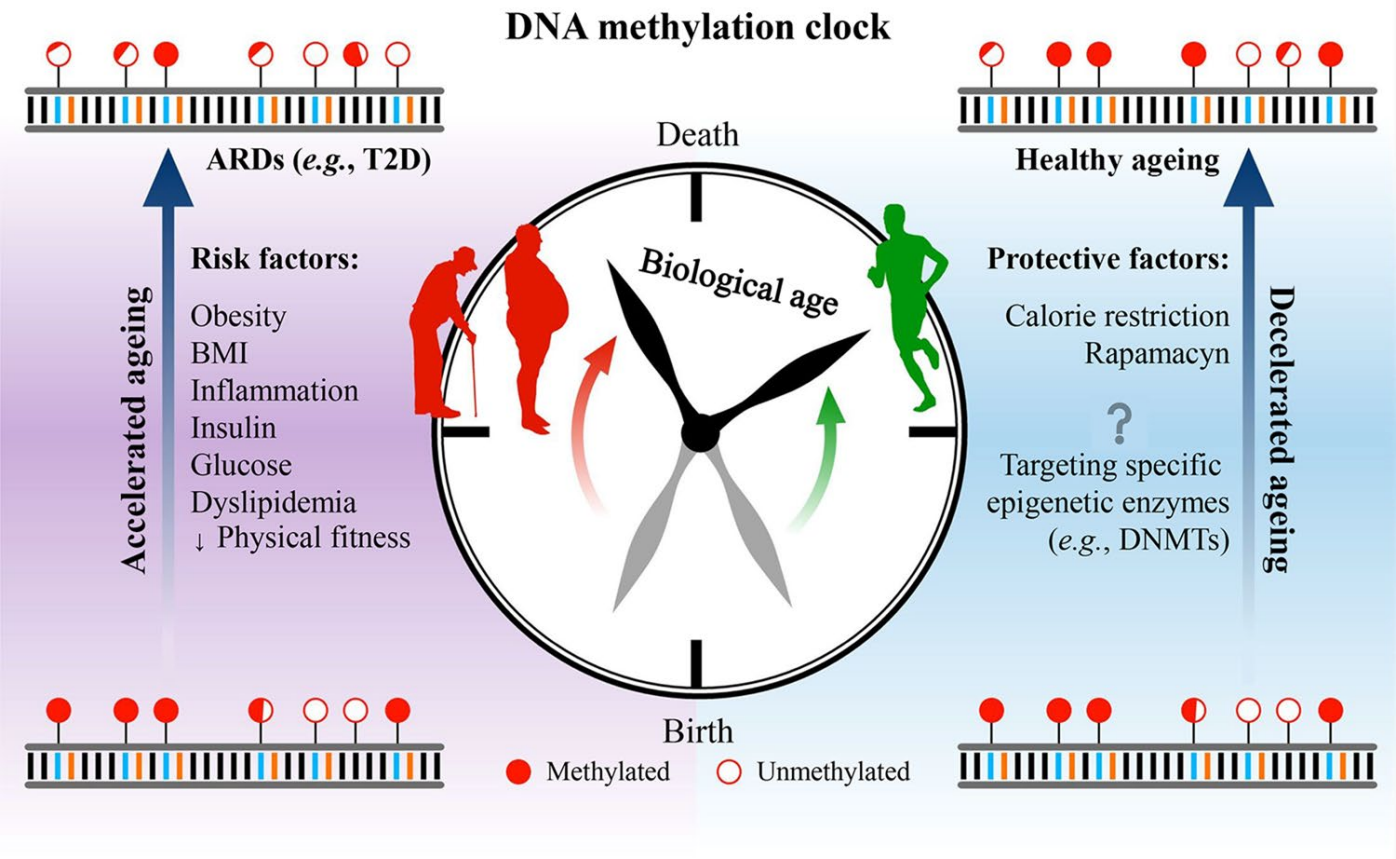
Schematic chart of a DNA methylation clock throughout the human lifetime. The difference between DNA methylation age and chronological age (i.e., the time elapsed since birth) reflects biological age and may be an indicator of ageing rate and health outcomes. ARDs, age-related diseases; T2D, type 2 diabetes

The effects of environmental stimuli on epigenetic ageing rates offer insight into how and why subjects with the same chronological age can experience dissimilar DNAm-age. The difference between DNAm-age and true chronological age reflects biological age (Δage), which is considered an indicator of human ageing rate and health outcomes [145, 166, 167]. Individuals can be categorized as (i) biologically old if their DNAm-age is higher than their chronological age; (ii) biologically young if the reverse is true; or, (iii) biologically concordant. Centenarians display a young biological age, while the risk of mortality is increased in biologically old subjects [168, 169]. Several lines of evidence support the notion of an accelerated DNAm-age resulting from advanced biological age. In addition, obesity, BMI, chronic systemic inflammation, T2D, NAFLD, and decreased physical fitness accelerate DNAm-age. Instead, interventions that promote longevity, such as CR and rapamycin, decelerate DNAm-age [145, 165, 170–172] (Fig. 3).

It should be noted that elevated BMI in middle-aged subjects but not in young adults (aged between 15 and 24) is positively correlated with accelerated epigenetic ageing. This evidence suggests that the acceleration of DNAm-age requires an extended period of exposure to obesity-related microenvironment. Interestingly, a positive correlation between BMI and accelerated epigenetic ageing has also been described during the next 25-year period in which the BMI increased. This suggests that advanced biological age is not only the product of the current BMI but also the increase in weight over time [173]. Additional experimental work will help clarify the causality of these relationships. Therefore, understanding DNAm-age molecular biology is essential in deciding how best using DNAm-age as a biomarker in biomedical research and in clinical medicine.

## Conclusion and future directions

Senescent cells increase during ageing and obesity and play a key role in T2D induction and exacerbation. Additionally, diabetes triggers a vicious cycle of development of senescent cells, which accelerate tissue damage and lead to diabetic complications. In the clinical setting, the causal nature of the relationship between cellular senescence and metabolic dysfunction has significant implications. Indeed, it has recently emerged that removing senescent cells is a successful therapeutic solution for reducing age-related metabolic diseases. Senescent cell targeting may be extended to obese and diabetic patients if such approach is confirmed to be effective in elderly humans and consequently, may have a significant impact on the prevention and treatment of T2D and its related complications.

Currently, significant attention is being given to therapeutic strategies that specifically target senescent cells, with several programmes now approaching human clinical studies. These include: (i) the use of senolytics (e.g., dasatinib + quercetin) to selectively kill senescent cells; and, (ii) the neutralization of the senescent cell secretome by inhibiting critical components of SASP (e.g., NF-kB, JAK, and mTOR). The first represents the best therapeutic opportunity as it permanently removes senescent cells, resulting in a persistent abolition of detrimental SASP effects. Given that modifications of the DNA methylome are necessary for both senescence initiation and maintenance of SASP, senolytic drugs that target specific epigenetic enzymes (e.g., DNMTs) have enormous potential to prevent age-related metabolic diseases.

Considering the reversible nature of epigenetic modifications, understanding the interplay between senescence and DNA methylation is a challenge for the immediate future. In the era of personalized medicine, it is necessary to move closer to an individualized view of healthy ageing and its relation to ARD prevention. As the elderly population continues to grow, these concerns are becoming increasingly more relevant.

## References

[1] Sierra F, Kohanski R (2015). Advances in geroscience.

[2] Tchkonia T, Kirkland JL (2018). Aging, cell senescence, and chronic disease: emerging therapeutic strategies. JAMA.

[3] Lancet T (2012). Chronic disease management in ageing populations. Lancet.

[4] Palmer AK, Kirkland JL (2016). Aging and adipose tissue: potential interventions for diabetes and regenerative medicine. Exp Gerontol.

[5] Stout MB, Justice JN, Nicklas BJ, Kirkland JL (2017). Physiological aging: links among adipose tissue dysfunction, diabetes, and frailty. Physiology (Bethesda).

[6] Sattar N, Rawshani A, Franzén S (2019). Age at diagnosis of type 2 diabetes mellitus and associations with cardiovascular and mortality risks. Circulation.

[7] García-Jiménez C, Gutiérrez-Salmerón M, Chocarro-Calvo A, García-Martinez JM, Castaño A, De la Vieja A (2016). From obesity to diabetes and cancer: epidemiological links and role of therapies. Br J Cancer.

[8] Fiory F, Spinelli R, Raciti GA (2017). Targetting PED/PEA-15 for diabetes treatment. Expert Opin Ther Targets.

[9] De Felice FG, Ferreira ST (2014). Inflammation, defective insulin signaling, and mitochondrial dysfunction as common molecular denominators connecting type 2 diabetes to Alzheimer disease. Diabetes.

[10] Franceschi C, Garagnani P, Morsiani C (2018). The continuum of aging and age-related diseases: common mechanisms but different rates. Front Med (Lausanne).

[11] Palmer AK, Gustafson B, Kirkland JL, Smith U (2019). Cellular senescence: at the nexus between ageing and diabetes. Diabetologia.

[12] Burton DGA, Faragher RGA (2018). Obesity and type-2 diabetes as inducers of premature cellular senescence and ageing. Biogerontology.

[13] Tchkonia T, Morbeck DE, von Zglinicki T (2010). Fat tissue, aging, and cellular senescence. Aging Cell.

[14] Stout MB, Tchkonia T, Kirkland JL, Fantuzzi G, Braunschweig C (2014). The aging adipose organ: lipid redistribution, inflammation, and cellular senescence. Adipose tissue and adipokines in health and disease. Nutrition and Health.

[15] Tchkonia T, Thomou T, Zhu Y, Karagiannides I, Pothoulakis C, Jensen MD, Kirkland JL (2013). Mechanisms and metabolic implications of regional differences among fat depots. Cell Metab.

[16] Tchkonia T, Tchoukalova YD, Giorgadze N (2005). Abundance of two human preadipocyte subtypes with distinct capacities for replication, adipogenesis, and apoptosis varies among fat depots. Am J Physiol Endocrinol Metab.

[17] Longo M, Zatterale F, Naderi J (2019). Adipose tissue dysfunction as determinant of obesity-associated metabolic complications. Int J Mol Sci.

[18] Hammarstedt A, Gogg S, Hedjazifar S, Nerstedt A, Smith U (2018). Impaired adipogenesis and dysfunctional adipose tissue in human hypertrophic obesity. Physiol Rev.

[19] Goodpaster BH, Krishnaswami S, Harris TB (2005). Obesity, regional body fat distribution, and the metabolic syndrome in older men and women. Arch Intern Med.

[20] Goodpaster BH, Krishnaswami S, Resnick H (2003). Association between regional adipose tissue distribution and both type 2 diabetes and impaired glucose tolerance in elderly men and women. Diabetes Care.

[21] Kyle UG, Genton L, Hans D, Karsegard VL, Michel JP, Slosman DO, Pichard C (2001). Total body mass, fat mass, fat-free mass, and skeletal muscle in older people: cross-sectional differences in 60-year-old persons. J Am Geriatr Soc.

[22] Kyle UG, Genton L, Slosman DO, Pichard C (2001). Fat-free and fat mass percentiles in 5225 healthy subjects aged 15 to 98 years. Nutrition.

[23] Kuk JL, Saunders TJ, Davidson LE, Ross R (2009). Age-related changes in total and regional fat distribution. Ageing Res Rev.

[24] Corrales P, Martín-Taboada M, Medina-Gomez G (2019). The risk of jiggly fat in aging. Aging (Albany NY).

[25] Fontana L, Klein S (2007). Aging, adiposity, and calorie restriction. JAMA.

[26] Most J, Tosti V, Redman LM, Fontana L (2017). Calorie restriction in humans: an update. Ageing Res Rev.

[27] Thompson D, Karpe F, Lafontan M, Frayn K (2012). Physical activity and exercise in the regulation of human adipose tissue physiology. Physiol Rev.

[28] Bays HE, Laferrère B, Dixon J (2009). Adiposopathy and Bariatric Surgery Working Group. Adiposopathy and bariatric surgery: is ‘sick fat’ a surgical disease?. Int J Clin Pract.

[29] López-Otín C, Blasco MA, Partridge L, Serrano M, Kroemer G (2013). The hallmarks of aging. Cell.

[30] Lakowa N, Trieu N, Flehmig G (2015). Telomere length differences between subcutaneous and visceral adipose tissue in humans. Biochem Biophys Res Commun.

[31] Schipper BM, Marra KG, Zhang W, Donnenberg AD, Rubin JP (2008). Regional anatomic and age effects on cell function of human adipose-derived stem cells. Ann Plast Surg.

[32] Fajas L (2003). Adipogenesis: a cross-talk between cell proliferation and cell differentiation. Ann Med.

[33] Cinti S (2002). Adipocyte differentiation and transdifferentiation: plasticity of the adipose organ. J Endocrinol Invest.

[34] Caso G, McNurlan MA, Mileva I, Zemlyak A, Mynarcik DC, Gelato MC (2013). Peripheral fat loss and decline in adipogenesis in older humans. Metabolism.

[35] Sepe A, Tchkonia T, Thomou T, Zamboni M, Kirkland JL (2011). Aging and regional differences in fat cell progenitors—a mini-review. Gerontology.

[36] Kirkland JL, Tchkonia T, Pirtskhalava T, Han J, Karagiannides I (2002). Adipogenesis and aging: does aging make fat go MAD?. Exp Gerontol.

[37] Ferrucci L, Fabbri E (2018). Inflammageing: chronic inflammation in ageing, cardiovascular disease, and frailty. Nat Rev Cardiol.

[38] Spranger J, Kroke A, Möhlig M (2003). Inflammatory cytokines and the risk to develop type 2 diabetes: results of the prospective population-based European Prospective Investigation into Cancer and Nutrition (EPIC)-Potsdam Study. Diabetes.

[39] Tchkonia T, Pirtskhalava T, Thomou T (2007). Increased TNFalpha and CCAAT/enhancer-binding protein homologous protein with aging predispose preadipocytes to resist adipogenesis. Am J Physiol Endocrinol Metab.

[40] Skurk T, Alberti-Huber C, Herder C, Hauner H (2007). Relationship between adipocyte size and adipokine expression and secretion. J Clin Endocrinol Metab.

[41] Martinez O, de Victoria E, Xu X, Koska J, Francisco AM, Scalise M, Ferrante AW, Krakoff J (2009). Macrophage content in subcutaneous adipose tissue: associations with adiposity, age, inflammatory markers, and whole-body insulin action in healthy Pima Indians. Diabetes.

[42] Freund A, Orjalo AV, Desprez PY, Campisi J (2010). Inflammatory networks during cellular senescence: causes and consequences. Trends Mol Med.

[43] Trabucco SE, Zhang H (2016). Finding Shangri-La: limiting the impact of senescence on aging. Cell Stem Cell.

[44] Xu M, Palmer AK, Ding H (2015). Targeting senescent cells enhances adipogenesis and metabolic function in old age. Elife.

[45] Xu M, Tchkonia T, Ding H (2015). JAK inhibition alleviates the cellular senescence-associated secretory phenotype and frailty in old age. Proc Natl Acad Sci USA.

[46] Nelson G, Wordsworth J, Wang C, Jurk D, Lawless C, Martin-Ruiz C, von Zglinicki T (2012). A senescent cell bystander effect: senescence-induced senescence. Aging Cell.

[47] da Silva PFL, Ogrodnik M, Kucheryavenko O (2019). The bystander effect contributes to the accumulation of senescent cells in vivo. Aging Cell.

[48] Xu M, Pirtskhalava T, Farr JN (2018). Senolytics improve physical function and increase lifespan in old age. Nat Med.

[49] Roos CM, Zhang B, Palmer AK (2016). Chronic senolytic treatment alleviates established vasomotor dysfunction in aged or atherosclerotic mice. Aging Cell.

[50] Farr JN, Xu M, Weivoda MM (2017). Targeting cellular senescence prevents age-related bone loss in mice. Nat Med.

[51] Lewis-McDougall FC, Ruchaya PJ (2019). Aged-senescent cells contribute to impaired heart regeneration. Aging Cell.

[52] Schafer MJ, White TA, Evans G (2016). Exercise prevents diet-induced cellular senescence in adipose tissue. Diabetes.

[53] Palmer AK, Xu M, Zhu Y, Pirtskhalava T (2019). Targeting senescent cells alleviates obesity-induced metabolic dysfunction. Aging Cell.

[54] Ogrodnik M, Zhu Y, Langhi LGP (2019). Obesity-induced cellular senescence drives anxiety and impairs neurogenesis. Cell Metab.

[55] Fuhrmann-Stroissnigg H, Ling YY (2017). Identification of HSP90 inhibitors as a novel class of senolytics. Nat Commun.

[56] Schafer MJ, White TA, Iijima K (2017). Cellular senescence mediates fibrotic pulmonary disease. Nat Commun.

[57] Hickson LJ, Langhi Prata LGP, Bobart SA (2020). Senolytics decrease senescent cells in humans: preliminary report from a clinical trial of Dasatinib plus Quercetin in individuals with diabetic kidney disease. EBioMedicine.

[58] Alt EU, Senst C, Murthy SN (2012). Aging alters tissue resident mesenchymal stem cell properties. Stem Cell Res.

[59] Karagiannides I, Tchkonia T, Dobson DE (2001). Altered expression of C/EBP family members results in decreased adipogenesis with aging. Am J Physiol Regul Integr Comp Physiol.

[60] Guo W, Pirtskhalava T, Tchkonia T, Xie W (2007). Aging results in paradoxical susceptibility of fat cell progenitors to lipotoxicity. Am J Physiol Endocrinol Metab.

[61] Cartwright MJ, Tchkonia T, Kirkland JL (2007). Aging in adipocytes: potential impact of inherent, depot-specific mechanisms. Exp Gerontol.

[62] Longo M, Spinelli R, D'Esposito V (2016). Pathologic endoplasmic reticulum stress induced by glucotoxic insults inhibits adipocyte differentiation and induces an inflammatory phenotype. Biochim Biophys Acta.

[63] Pirone L, Smaldone G, Spinelli R (2019). KCTD1: a novel modulator of adipogenesis through the interaction with the transcription factor AP2α. Biochim Biophys Acta Mol Cell Biol Lipids.

[64] Argmann C, Dobrin R, Heikkinen S (2009). Ppargamma2 is a key driver of longevity in the mouse. PLoS Genet.

[65] Tchkonia T, Lenburg M, Thomou T (2007). Identification of depot-specific human fat cell progenitors through distinct expression profiles and developmental gene patterns. Am J Physiol Endocrinol Metab.

[66] Keller M, Hopp L, Liu X (2016). Genome-wide DNA promoter methylation and transcriptome analysis in human adipose tissue unravels novel candidate genes for obesity. Mol Metab.

[67] Mau T, Yung R (2018). Adipose tissue inflammation in aging. Exp Gerontol.

[68] Ghosh AK, Garg SK, Mau T, O'Brien M, Liu J, Yung R (2015). Elevated endoplasmic reticulum stress response contributes to adipose tissue inflammation in aging. Jerontol A Biol Sci Med Sci.

[69] Ghosh AK, Mau T, O'Brien M, Garg S, Yung R (2016). Impaired autophagy activity is linked to elevated ER-stress and inflammation in aging adipose tissue. Aging (Albany NY).

[70] Lumeng CN, Liu J, Geletka L (2011). Aging is associated with an increase in T cells and inflammatory macrophages in visceral adipose tissue. J Immunol.

[71] Garg SK, Delaney C, Toubai T, Ghosh A, Reddy P, Banerjee R, Yung R (2014). Aging is associated with increased regulatory T-cell function. Aging Cell.

[72] Sharma S, Dominguez AL, Lustgarten J (2006). High accumulation of T regulatory cells prevents the activation of immune responses in aged animals. J Immunol.

[73] Bapat SP, Myoung Suh J, Fang S (2015). Depletion of fat-resident Treg cells prevents age-associated insulin resistance. Nature.

[74] McHugh D, Gil J (2018). Senescence and aging: causes, consequences, and therapeutic avenues. J Cell Biol.

[75] Herranz N, Gil J (2018). Mechanisms and functions of cellular senescence. J Clin Invest.

[76] Childs BG, Durik M, Baker DJ, van Deursen JM (2015). Cellular senescence in aging and age-related disease: from mechanisms to therapy. Nat Med.

[77] Charar C, Gruenbaum Y (2017). Lamins and metabolism. Clin Sci (Lond).

[78] Baker DJ, Weaver RL, van Deursen JM (2013). p21 both attenuates and drives senescence and aging in BubR1 progeroid mice. Cell Rep.

[79] Muñoz-Espín D, Serrano M (2014). Cellular senescence: from physiology to pathology. Nat Rev Mol Cell Biol.

[80] Kuilman T, Michaloglou C, Mooi WJ, Peeper DS (2010). The essence of senescence. Genes Dev.

[81] Hernandez-Segura A, Nehme J, Demaria M (2018). Hallmarks of cellular senescence. Trends Cell Biol.

[82] Faget DV, Ren Q, Stewart SA (2019). Unmasking senescence: context-dependent effects of SASP in cancer. Nat Rev Cancer.

[83] Gustafson B, Nerstedt A, Smith U (2019). Reduced subcutaneous adipogenesis in human hypertrophic obesity is linked to senescent precursor cells. Nat Commun.

[84] Shah PP, Donahue G, Otte GL (2013). Lamin B1 depletion in senescent cells triggers large-scale changes in gene expression and the chromatin landscape. Genes Dev.

[85] Wiley CD, Flynn JM, Morrissey C (2017). Analysis of individual cells identifies cell-to-cell variability following induction of cellular senescence. Aging Cell.

[86] Ghosh AK, O'Brien M, Mau T, Qi N, Yung R (2019). Adipose tissue senescence and inflammation in aging is reversed by the Young Milieu. J Gerontol A Biol Sci Med Sci.

[87] Ahima RS (2009). Connecting obesity, aging and diabetes. Nat Med.

[88] Nilsson E, Ling C (2017). DNA methylation links genetics, fetal environment, and an unhealthy lifestyle to the development of type 2 diabetes. Clin Epigenetics.

[89] Parrillo L, Spinelli R, Nicolò A (2019). Nutritional factors, DNA methylation, and risk of type 2 diabetes and obesity: perspectives and challenges. Int J Mol Sci.

[90] Ungaro P, Mirra P, Oriente F (2012). Peroxisome proliferator-activated receptor-γ activation enhances insulin-stimulated glucose disposal by reducing ped/pea-15 gene expression in skeletal muscle cells: evidence for involvement of activator protein-1. J Biol Chem.

[91] Raciti GA, Spinelli R, Desiderio A (2017). Specific CpG hyper-methylation leads to Ankrd26 gene down-regulation in white adipose tissue of a mouse model of diet-induced obesity. Sci Rep.

[92] Parrillo L, Costa V, Raciti GA (2016). Hoxa5 undergoes dynamic DNA methylation and transcriptional repression in the adipose tissue of mice exposed to high-fat diet. Int J Obes (Lond).

[93] Desiderio A, Longo M, Parrillo L (2019). Epigenetic silencing of the ANKRD26 gene correlates to the pro-inflammatory profile and increased cardio-metabolic risk factors in human obesity. Clin Epigenetics.

[94] Minamino T, Orimo M, Shimizu I (2009). A crucial role for adipose tissue p53 in the regulation of insulin resistance. Nat Med.

[95] Krstic J, Reinisch I, Schupp M, Schulz TJ, Prokesch A (2018). p53 Functions in adipose tissue metabolism and homeostasis. Int J Mol Sci.

[96] Vergoni B, Cornejo PJ, Gilleron J (2016). DNA damage and the activation of the p53 pathway mediate alterations in metabolic and secretory functions of adipocytes. Diabetes.

[97] Justice JN, Gregory H, Tchkonia T, LeBrasseur NK, Kirkland JL, Kritchevsky SB, Nicklas BJ (2018). Cellular senescence biomarker p16INK4a+ cell burden in thigh adipose is associated with poor physical function in older women. J Gerontol A Biol Sci Med Sci.

[98] Hall JA, Dominy JE, Lee Y, Puigserver P (2013). The sirtuin family's role in aging and age-associated pathologies. J Clin Invest.

[99] Huynh FK, Hershberger KA, Hirschey MD (2013). Targeting sirtuins for the treatment of diabetes. Diabetes Manag (Lond).

[100] Xu F, Gao Z, Zhang J (2010). Lack of SIRT1 (Mammalian Sirtuin 1) activity leads to liver steatosis in the SIRT1+/- mice: a role of lipid mobilization and inflammation. Endocrinology.

[101] Chalkiadaki A, Guarente L (2012). High-fat diet triggers inflammation-induced cleavage of SIRT1 in adipose tissue to promote metabolic dysfunction. Cell Metab.

[102] Cho KW, Lumeng CN (2011). SirT1: a guardian at the gates of adipose tissue inflammation. Diabetes.

[103] Hui X, Zhang M, Gu P (2017). Adipocyte SIRT1 controls systemic insulin sensitivity by modulating macrophages in adipose tissue. EMBO Rep.

[104] Ka SO, Song MY, Bae EJ, Park BH (2015). Myeloid SIRT1 regulates macrophage infiltration and insulin sensitivity in mice fed a high-fat diet. J Endocrinol.

[105] Perrini S, Porro S, Nigro P (2020). Reduced SIRT1 and SIRT2 expression promotes adipogenesis of human visceral adipose stem cells and associates with accumulation of visceral fat in human obesity. Int J Obes (Lond).

[106] Song YS, Lee SK, Jang YJ (2013). Association between low SIRT1 expression in visceral and subcutaneous adipose tissues and metabolic abnormalities in women with obesity and type 2 diabetes. Diabetes Res Clin Pract.

[107] Gillum MP, Kotas ME, Erion DM (2011). SirT1 regulates adipose tissue inflammation. Diabetes.

[108] Kursawe R, Dixit VD, Scherer PE (2016). A role of the inflammasome in the low storage capacity of the abdominal subcutaneous adipose tissue in obese adolescents. Diabetes.

[109] Yoshizaki T, Milne JC, Imamura T (2009). SIRT1 exerts anti-inflammatory effects and improves insulin sensitivity in adipocytes. Mol Cell Biol.

[110] Fang J, Ianni A, Smolka C (2017). Sirt7 promotes adipogenesis in the mouse by inhibiting autocatalytic activation of Sirt1. Proc Natl Acad Sci USA.

[111] Langley E, Pearson M, Faretta M (2002). Human SIR2 deacetylates p53 and antagonizes PML/p53-induced cellular senescence. EMBO J.

[112] Amano H, Chaudhury A, Rodriguez-Aguayo C (2019). Telomere dysfunction induces sirtuin repression that drives telomere-dependent disease. Cell Metab.

[113] Amano H, Sahin E (2019). Telomeres and sirtuins: at the end we meet again. Mol Cell Oncol.

[114] Khanh VC, Zulkifli AF, Tokunaga C, Yamashita T, Hiramatsu Y, Ohneda O (2018). Aging impairs beige adipocyte differentiation of mesenchymal stem cells via the reduced expression of Sirtuin 1. Biochem Biophys Res Commun.

[115] Rufini A, Tucci P, Celardo I, Melino G (2013). Senescence and aging: the critical roles of p53. Oncogene.

[116] Mitterberger MC, Lechner S, Mattesich M, Zwerschke W (2014). Adipogenic differentiation is impaired in replicative senescent human subcutaneous adipose-derived stromal/progenitor cells. J Gerontol A Biol Sci Med Sci.

[117] Oñate B, Vilahur G, Ferrer-Lorente R (2012). The subcutaneous adipose tissue reservoir of functionally active stem cells is reduced in obese patients. FASEB J.

[118] Lotta LA, Gulati P, Day FR (2019). Integrative genomic analysis implicates limited peripheral adipose storage capacity in the pathogenesis of human insulin resistance. Nat Genet.

[119] Longo M, Raciti GA, Zatterale F (2018). Epigenetic modifications of the Zfp/ZNF423 gene control murine adipogenic commitment and are dysregulated in human hypertrophic obesity. Diabetologia.

[120] Parrillo L, Spinelli R, Longo M (2020). Altered PTPRD DNA methylation associates with restricted adipogenesis in healthy first-degree relatives of T2D subjects. Epigenomics.

[121] Palmer AK, Tchkonia T, LeBrasseur NK, Chini EN, Xu M, Kirkland JL (2015). Cellular senescence in type 2 diabetes: a therapeutic opportunity. Diabetes.

[122] Strycharz J, Drzewoski J, Szemraj J, Sliwinska A (2017). Is p53 involved in tissue-specific insulin resistance formation?. Oxid Med Cell Longev.

[123] Berná G, Oliveras-López MJ, Jurado-Ruíz E, Tejedo J, Bedoya F, Soria B, Martín F (2014). Nutrigenetics and nutrigenomics insights into diabetes etiopathogenesis. Nutrients.

[124] Hannou SA, Wouters K, Paumelle R, Staels B (2014). Functional genomics of the CDKN2A/B locus in cardiovascular and metabolic disease: what have we learned from GWASs?. Trends Endocrinol Metab.

[125] Leslie R, O’Donnell CJ, Johnson AD (2014). GRASP: analysis of genotype-phenotype results from 1,390 genome-wide association studies and corresponding open access database. Bioinformatics.

[126] Strazhesko I, Tkacheva O, Boytsov S (2015). Association of insulin resistance, arterial stiffness and telomere length in adults free of cardiovascular diseases. PLoS ONE.

[127] Gardner JP, Li S, Srinivasan SR (2005). Rise in insulin resistance is associated with escalated telomere attrition. Circulation.

[128] Testa R, Olivieri F, Sirolla C (2011). Leukocyte telomere length is associated with complications of type 2 diabetes mellitus. Diabet Med.

[129] Monickaraj F, Gokulakrishnan K, Prabu P (2012). Convergence of adipocyte hypertrophy, telomere shortening and hypoadiponectinemia in obese subjects and in patients with type 2 diabetes. Clin Biochem.

[130] Prattichizzo F, De Nigris V, La Sala L, Procopio AD, Olivieri F, Ceriello A (2016). "Inflammaging" as a druggable target: a senescence-associated secretory phenotype-centered view of type 2 diabetes. Oxid Med Cell Longev.

[131] Cramer C, Freisinger E, Jones RK (2010). Persistent high glucose concentrations alter the regenerative potential of mesenchymal stem cells. Stem Cells Dev.

[132] Yokoi T, Fukuo K, Yasuda O (2006). Apoptosis signal-regulating kinase 1 mediates cellular senescence induced by high glucose in endothelial cells. Diabetes.

[133] Ksiazek K, Passos JF, Olijslagers S, von Zglinicki T (2008). Mitochondrial dysfunction is a possible cause of accelerated senescence of mesothelial cells exposed to high glucose. Biochem Biophys Res Commun.

[134] Peppa M, Uribarri J, Vlassara H (2003). Glucose, advanced glycation end products, and diabetes complications: what is new and what works. Clin Diabetes.

[135] Nigro C, Leone A, Longo M (2019). Methylglyoxal accumulation de-regulates HoxA5 expression, thereby impairing angiogenesis in glyoxalase 1 knock-down mouse aortic endothelial cells. Biochim Biophys Acta Mol Basis Dis.

[136] Liu J, Huang K, Cai GY (2014). Receptor for advanced glycation end products promotes premature senescence of proximal tubular epithelial cells via activation of endoplasmic reticulum stress-dependent p21 signaling. Cell Signal.

[137] Tran D, Bergholz J, Zhang H (2014). Insulin-like growth factor-1 regulates the SIRT1-p53 pathway in cellular senescence. Aging Cell.

[138] Elzi DJ, Lai Y, Song M, Hakala K, Weintraub ST, Shiio Y (2012). Plasminogen activator inhibitor 1–insulin-like growth factor binding protein 3 cascade regulates stress-induced senescence. Proc Natl Acad Sci USA.

[139] Trayssac M, Hannun YA, Obeid LM (2018). Role of sphingolipids in senescence: implication in aging and age-related diseases. J Clin Invest.

[140] Verzola D, Gandolfo MT, Gaetani G (2008). Accelerated senescence in the kidneys of patients with type 2 diabetic nephropathy. Am J Physiol Renal Physiol.

[141] Kim SR, Jiang K, Ogrodnik M (2019). Increased renal cellular senescence in murine high-fat diet: effect of the senolytic drug quercetin. Transl Res.

[142] Pal S, Tyler JK (2016). Epigenetics and aging. Sci Adv.

[143] Hadad N, Masser DR, Blanco-Berdugo L, Stanford DR, Freeman WM (2019). Early-life DNA methylation profiles are indicative of age-related transcriptome changes. Epigenetics Chromatin.

[144] Desiderio A, Spinelli R, Ciccarelli M, Nigro C, Miele C, Beguinot F, Raciti GA (2016). Epigenetics: spotlight on type 2 diabetes and obesity. J Endocrinol Invest.

[145] Field AE, Robertson NA, Wang T, Havas A, Ideker T, Adams PD (2018). DNA methylation clocks in aging: categories, causes, and consequences. Mol Cell.

[146] Ciccarone F, Malavolta M, Calabrese R (2016). Age-dependent expression of DNMT1 and DNMT3B in PBMCs from a large European population enrolled in the MARK-AGE study. Aging Cell.

[147] Rönn T, Volkov P, Gillberg L (2015). Impact of age, BMI and HbA1c levels on the genome-wide DNA methylation and mRNA expression patterns in human adipose tissue and identification of epigenetic biomarkers in blood. Hum Mol Genet.

[148] Issa JP (2014). Aging and epigenetic drift: a vicious cycle. J Clin Invest.

[149] Sidler C, Kovalchuk O, Kovalchuk I (2017). Epigenetic regulation of cellular senescence and aging. Front Genet.

[150] Nacarelli T, Liu P, Zhang R (2016). Epigenetic Basis of Cellular Senescence and Its Implications in Aging. Genes (Basel).

[151] Cheng LQ, Zhang ZQ, Chen HZ, Liu DP (2017). Epigenetic regulation in cell senescence. J Mol Med (Berl).

[152] So AY, Jung JW, Lee S, Kim HS, Kang KS (2011). DNA methyltransferase controls stem cell aging by regulating BMI1 and EZH2 through microRNAs. PLoS ONE.

[153] Balakrishnan A, Guruprasad KP, Satyamoorthy K, Joshi MB (2018). Interleukin-6 determines protein stabilization of DNA methyltransferases and alters DNA promoter methylation of genes associated with insulin signaling and angiogenesis. Lab Invest.

[154] Gonzalez-Jaramillo V, Portilla-Fernandez E, Glisic M (2019). Epigenetics and inflammatory markers: a systematic review of the current evidence. Int J Inflam.

[155] Zannas AS, Jia M, Hafner K (2019). Epigenetic upregulation of FKBP5 by aging and stress contributes to NF-κB-driven inflammation and cardiovascular risk. Proc Natl Acad Sci USA.

[156] Lascar N, Brown J, Pattison H, Barnett AH, Bailey CJ, Bellary S (2018). Type 2 diabetes in adolescents and young adults. Lancet Diabetes Endocrinol.

[157] Horvath S (2013). DNA methylation age of human tissues and cell types. Genome Biol.

[158] Hannum G, Guinney J, Zhao L (2013). Genome-wide methylation profiles reveal quantitative views of human aging rates. Mol Cell.

[159] Bocklandt S, Lin W, Sehl ME, Sánchez FJ, Sinsheimer JS, Horvath S, Vilain E (2011). Epigenetic predictor of age. PLoS ONE.

[160] Huang Y, Yan J, Hou J, Fu X, Li L, Hou Y (2015). Developing a DNA methylation assay for human age prediction in blood and bloodstain. Forensic Sci Int Genet.

[161] Zbieć-Piekarska R, Spólnicka M, Kupiec T (2015). Development of a forensically useful age prediction method based on DNA methylation analysis. Forensic Sci Int Genet.

[162] Florath I, Butterbach K, Müller H, Bewerunge-Hudler M, Brenner H (2014). Cross-sectional and longitudinal changes in DNA methylation with age: an epigenome-wide analysis revealing over 60 novel age-associated CpG sites. Hum Mol Genet.

[163] Nwanaji-Enwerem JC, Weisskopf MG, Baccarelli AA (2018). Multi-tissue DNA methylation age: Molecular relationships and perspectives for advancing biomarker utility. Ageing Res Rev.

[164] Sturm G, Cardenas A, Bind MA (2019). Human aging DNA methylation signatures are conserved but accelerated in cultured fibroblasts. Epigenetics.

[165] Horvath S, Erhart W, Brosch M (2014). Obesity accelerates epigenetic aging of human liver. Proc Natl Acad Sci USA.

[166] Jones MJ, Goodman SJ, Kobor MS (2015). DNA methylation and healthy human aging. Aging Cell.

[167] Gentilini D, Mari D, Castaldi D (2013). Role of epigenetics in human aging and longevity: genome-wide DNA methylation profile in centenarians and centenarians' offspring. Age (Dordr).

[168] Marioni RE, Shah S, McRae AF (2015). DNA methylation age of blood predicts all-cause mortality in later life. Genome Biol.

[169] Cole JJ, Robertson NA, Rather MI (2017). Diverse interventions that extend mouse lifespan suppress shared age-associated epigenetic changes at critical gene regulatory regions. Genome Biol.

[170] Stevenson AJ, McCartney DL, Harris SE (2018). Trajectories of inflammatory biomarkers over the eighth decade and their associations with immune cell profiles and epigenetic ageing. Clin Epigenetics.

[171] Franceschi C, Garagnani P, Parini P, Giuliani C, Santoro A (2018). Inflammaging: a new immune-metabolic viewpoint for age-related diseases. Nat Rev Endocrinol.

[172] Bacos K, Gillberg L, Volkov P (2016). Blood-based biomarkers of age-associated epigenetic changes in human islets associate with insulin secretion and diabetes. Nat Commun.

[173] Nevalainen T, Kananen L, Marttila S (2017). Obesity accelerates epigenetic aging in middle-aged but not in elderly individuals. Clin Epigenetics.

